# Automated elaborate resection planning for bone tumor surgery

**DOI:** 10.1007/s11548-022-02763-4

**Published:** 2022-11-01

**Authors:** Dave Hill, Tom Williamson, Chow Yin Lai, Martin Leary, Milan Brandt, Peter Choong

**Affiliations:** 1 Centre for Additive Manufacturing, School of Engineering, RMIT University, 58 Cardigan St, Carlton, 3001 Australia; 2 Department of Electronic and Electrical Engineering, University College London, Malet Place and Torrington Place, Roberts Building, Level 7, London, WC1E 7JE UK; 3 Department of Surgery, University of Melbourne, Level 2, Clinical Sciences Building, 29 Regent Street, Fitzroy, 3065 Australia

**Keywords:** Bone tumors, Surgical planning, Automated planning, Orthopedic oncology, Robotic surgery

## Abstract

**Purpose:**

Planning for bone tumor resection surgery is a technically demanding and time-consuming task, reliant on manual positioning of planar cuts in a virtual space. More elaborate cutting approaches may be possible through the use of surgical robots or patient-specific instruments; however, methods for preparing such a resection plan must be developed.

**Methods:**

This work describes an automated approach for generating conformal bone tumor resection plans, where the resection geometry is defined by the convex hull of the tumor, and a focal point. The resection geometry is optimized using particle swarm, where the volume of healthy bone collaterally resected with the tumor is minimized. The approach was compared to manually prepared planar resection plans from an experienced surgeon for 20 tumor cases.

**Results:**

It was found that algorithm-generated hull-type resections greatly reduced the volume of collaterally resected healthy bone. The hull-type resections resulted in statistically significant improvements compared to the manual approach (paired *t *test, *p* < 0.001).

**Conclusions:**

The described approach has potential to improve patient outcomes by reducing the volume of healthy bone collaterally resected with the tumor and preserving nearby critical anatomy.

**Supplementary Information:**

The online version contains supplementary material available at 10.1007/s11548-022-02763-4.

## Introduction

Bone tumors are a rare condition which can affect people of all ages but is more frequently seen in people aged 10–25 years, and people over 50 years [1]. In the USA, bone tumors account for approximately 3–5% of cancer cases in children [2], and in Australia about 250 people are diagnosed with primary bone cancer each year [1]**.** The main aim of bone tumor surgery is to completely excise the tumor and ensure no diseased cells remain in the surgical site. It is standard practice to remove bone tumors *en-bloc* (in one piece) along with a cuff of collateral healthy tissue (margin) to decrease the likelihood of local recurrence of the disease. The selected surgical approach depends on the size and location of the tumor, its proximity to critical anatomical structures, and the possibility of preserving and reconstructing the affected limb [3].

Modern imaging and planning software affords the surgeon more detail during preoperative planning, assisting in preserving anatomical structures [4, 5] and significantly decreasing the likelihood of intra-lesional resections [6]. The individual nature of every tumor means preoperative planning is still a technically demanding and time-consuming process, with the time taken to generate a plan associated with the complexity of the case, a surgeon’s experience with similar cases, and their familiarity with virtual planning software [7].

Automated planning systems have been tested in orthopedic oncology and similar fields. Carrillo et al. [8] developed a genetic algorithm to plan corrective osteotomies for patients with curved forearm deformities, generating solutions that include the configuration of the osteotomy plane, fixation plates, and screws. The solutions proposed by the genetic algorithm were deemed clinically feasible, and in 50% of cases were considered improvements upon a manually generated plan for the same case. Zhang et al*.* [9] proposed a semi-automated approach to generate bone tumor resection plans where a surgeon defines a curved danger region around the tumor and the number of cutting planes to be used, with an algorithm then optimizing the configuration of the cutting planes for the minimum amount of healthy bone to be taken with the tumor. Defining a danger region limits the algorithm-generated resection plans such that they do not intersect with potentially diseased tissue, ensuring a safe margin and the entire tumor is removed from the operative site.

Other recent research in this field [10] advances the autonomy and consistency of algorithm-generated resections and reports that a greater volume of healthy bone can be preserved through additional cutting planes, but there are diminishing returns on this value when using more than five planar cuts.

While these planning algorithms assist in preserving healthy bone, the resections themselves are limited to planar cuts only. More elaborate resections which conform to the surface of the tumor may have greater potential to preserve healthy bone and avoid collateral anatomy; however, increasing the complexity of the tool path would necessitate finer and more exact control over the position and alignment of the cutting tool, which could be achieved through patient-specific cutting guides, or surgical robots.

Patient-specific cutting guides can be used where intricate cuts are required to avoid critical anatomy [11], or to ensure a reconstructive solution and the prepared surgical site will match geometry [12, 13]. Although cutting guides can facilitate curvilinear tool paths, their application still tends to be for the precise alignment of planar cuts.

There is a long history of the use of robotics in arthroplasty and orthopedic procedures, and the potential benefit of robotics is commonly evaluated by comparing to the existing manual approach. Often a procedure or technique is performed by both surgeons and a robot [14–17], and the subsequent technical evaluation considers factors such as the relative linear and angular precision of a cut as performed by the robot and the unassisted surgeon. This process of testing the capabilities of a surgical robot through incremental integration into well-established procedures means the primary contribution of the robot is reduced to only improving the consistency of the existing approach, essentially limiting the functionality of the robot to the same processes the surgeon was already capable of performing, albeit with greater precision and accuracy.

Various studies have shown that surgical robots are capable of performing intraoperative tasks with a high degree of precision [16–19], as well as following complex non-planar and curvilinear paths for approaches that would not be possible to perform accurately or safely freehand. Khan et al*.* evaluated the deviation from a planned multi-planar osteotomy, comparing the accuracy of freehand unassisted sawing against the same cuts performed with robotic assistance. After cutting operations, virtual models of the resected volumes were compared to the original plan, with the robot-assisted surgery demonstrating mean improvements to the pitch (7.9°), roll (4.6°), and linear deviation (7.8 mm) of a cutting plane compared to the freehand cutting [16]. Research by Cattin et al*.* [20] demonstrates a precise sinusoidal osteotomy in a mandible, produced via robotically controlled laser ablation of bone along a curvilinear tool path, a task that would be extremely challenging to accurately reproduce without robotic assistance.

In the context of bone tumor surgery, reconsidering the procedure without limiting the capability of the surgical robot may facilitate more elaborate resection geometries. A curvilinear tool path, or a more complex tool path analogous to a ruled surface [21], could closely follow the tumor margin or a defined danger region. This may allow for greater preservation of healthy bone and critical anatomy, with a resulting complex void that could be reconstructed using an additively manufactured patient-specific implant [22].

In order to improve upon the current manual process of resection planning and examine the potential benefits of non-planar resection plans, this paper investigates an automated approach to preoperative planning for *en-bloc* bone tumor surgery by generating an elaborate resection plan determined by the tumor geometry. It was hypothesized that generating a resection plan using the convex hull of the tumor would result in less healthy bone collaterally removed than a standard manually generated planar resection for the same tumor. The scope of this research extends only to generation of the resection geometries used as preoperative plans. The implementation of the described approach, including clinical relevance and reproducibility, is the subject of complimentary research.

## Materials and methods

The approach generates elaborate resection plans by rotating and translating 3D bone and tumor data about Cartesian coordinate space, then generating a geometry (comparable to a ruled surface) based on the features and position of the bone and tumor surface mesh.

With review board permission (approval ID: LNR/18/SVHM/21), de-identified data for 20 tumor cases were sourced from a retrospective study. All cases were primary malignant monolithic tumors around the knee (11 osteosarcoma, 9 chondrosarcoma), in either the proximal tibia (8) or distal femur (12), with at least one set of magnetic resonance or computed tomography image data visualizing the condylar surface nearest the diseased site. Using open-source software (MITK, DKFZ, Germany), the authors manually segmented separate bone and tumor models by tracing each slice of the 3D image, then merging the slices into surface meshes with an in-built marching cubes algorithm and smoothing function. The cases included a diverse range of the possible sizes and positions of tumors around the knee (visualizations in Electronic Supplementary Material).

**Fig. 1:**
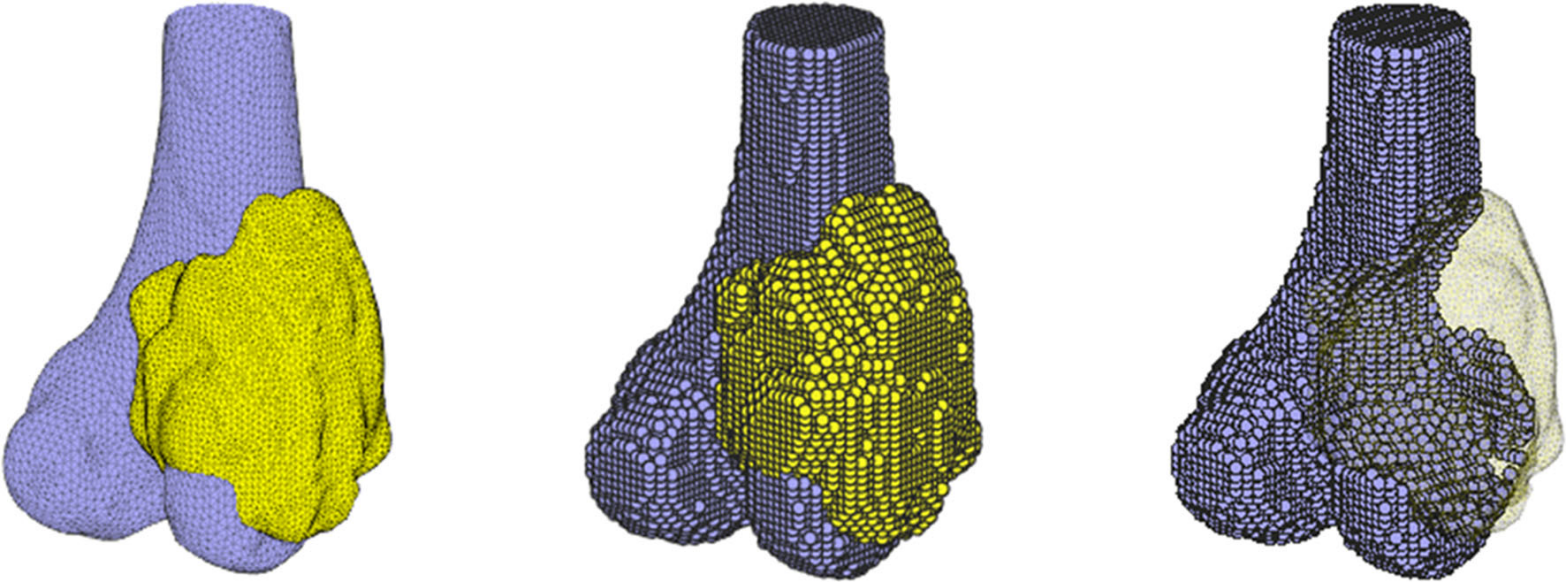
Surface (left) and voxel model (middle) of bone and tumor, showing healthy bone outside tumor (right)

The segmented bone and tumor surface mesh models were imported into MATLAB (MATLAB R2018a, The MathWorks Inc., USA), and set inside a grid of uniformly spaced points (2 × 2 × 2 mm cubic spacing, coordinates set at cube centers) spanning the 3D bounds of the surface meshes. Using a ray intersection function [23], the position of each grid point was calculated with respect to the bone and tumor surface meshes; grid points outside both meshes were discarded, while those inside either surface mesh were retained, resulting in homogenous volumetric models for the bone and tumor. The bone volumetric points (voxels) inside the tumor surface mesh were discarded, leaving a voxel array of healthy bone, a voxel array of the tumor, along with two surface mesh models (Fig. 1). All four sets of points were translated such that the centroid of the tumor surface mesh was coincident with the origin (*x*_0_*, y*_0_*, z*_0_), retaining the original alignment of the segmented bone and tumor scan data.

Three elaborate resection geometries (RGs) were investigated, based on computing the 3D convex hull of the tumor surface mesh and a focal point, resulting in a quasi-conical surface. The surface fully envelops the tumor along with some quantity of healthy bone voxels, equivalent to the volume of collaterally resected bone. For all cases, to simplify the differences in margin requirements and examine the consistency and capability of the algorithm, the tumor surface mesh was assumed to include a cuff of healthy tissue. The following sections describe sets of input parameters which can be optimized for each of the defined RGs.

### Conical hull

The conical hull RG is the 3D convex hull of the tumor surface mesh and a focal point set some distance from the tumor. The RG is generated by rotating the bone and tumor meshes about the initial coordinate space, applying a translation in the new local coordinates, then taking the 3D convex hull of the tumor surface and focal point in the updated position.

**Fig. 2:**
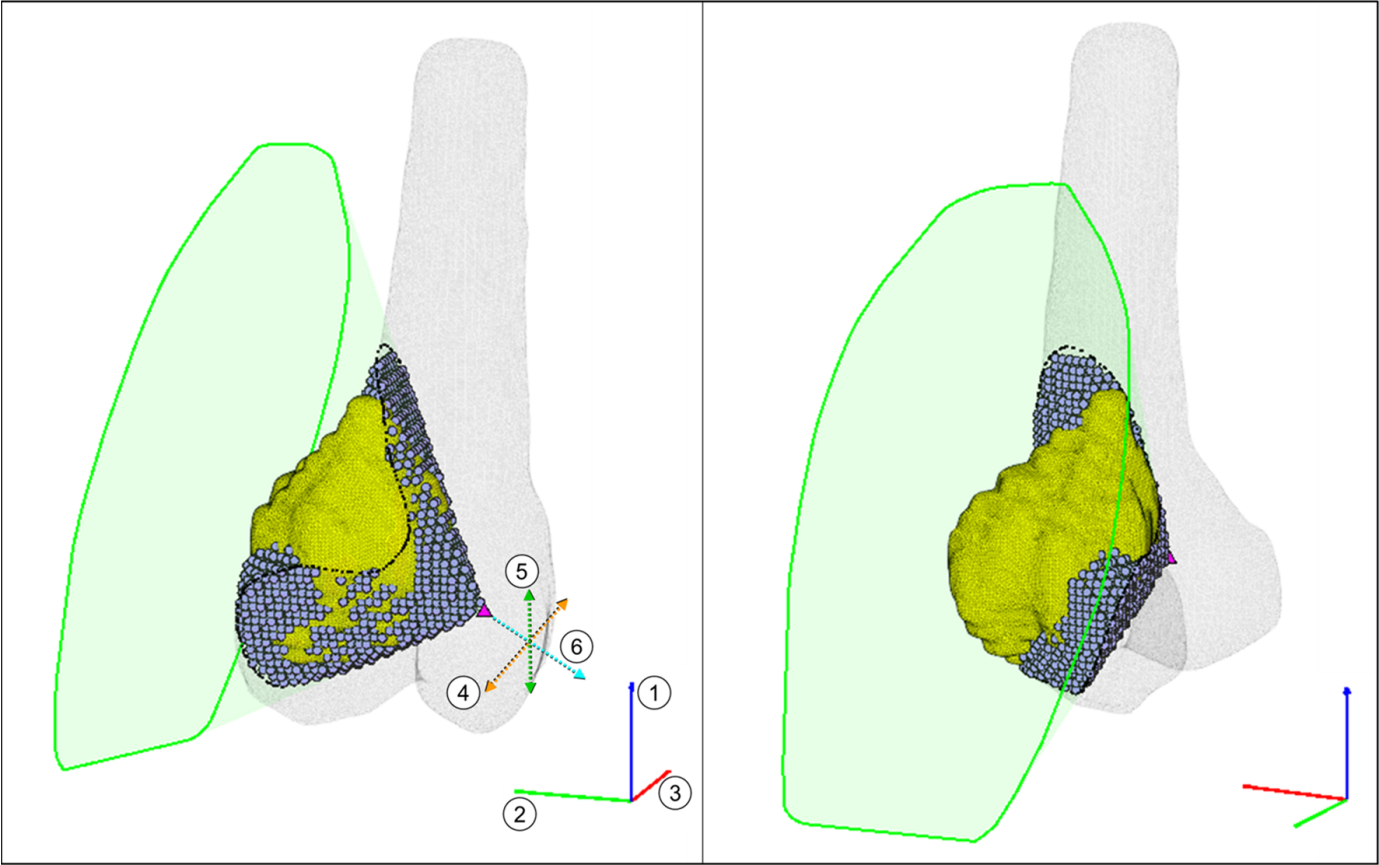
Conical hull resection (case 9) showing RG comprised of focal point (magenta triangle) and conical profile around tumor (green spline), and collaterally resected bone (gray circles). Base *xyz* coordinates indicated by red–green–blue (RGB) axes, with possible axes of rotation (1–3). Translation in tumor local coordinate system indicated by dashed orange, green, and cyan lines (4–6)

Three separate rotations are applied to realign the meshes about the $z$, $y$, and $x$ axes. The bone and tumor are then shifted along the local $x$- and $y$-axes, no further than the most distant points of the tumor mesh in each axis, such that the origin remains within the $x$ and $y$ bounds of the tumor. The meshes are translated along the local $z$-axis, such that the lowest point of the tumor is set some distance above the origin. The convex hull of the tumor mesh and the origin is computed, and all lines connecting the focal point to the tumor 3D convex hull are extended beyond the most distant point within the hull profile (tumor or bone) to ensure the resected volume can be removed. The points comprising the conical hull are transformed back to the initial coordinate space, resulting in a focal point and a spline of connecting points in space. The volume of the resection was calculated by counting the number of healthy bone voxels within the 3D convex hull of this geometry, indicated by Eq. (1),

1 $$ B_{xyz} \in {\text{inhull}}\left( {T_{xyz} ,FP} \right) $$

where inhull() determines whether points are inside or outside a convex hull set, $B_{xyz}$ is the bone points, $T_{xyz}$ is the surface mesh points, and $FP$ is the focal point. The RG is defined by six input values; three for the $z$, $y$, and $x$ rotations, and three for the $x$, $y$, and $z$ translation of the focal point in the rotated coordinate system (Fig. 2). Any input between the bounds of each variable (Table 1) will produce a conical hull resection.

**Table 1:** Conical hull resection value bounds and purpose

Variable	Purpose	Lower bound	Upper bound
1	Rotation about base *z*-axis	− 180°	+ 180°
2	Rotation about base *y*-axis	− 180°	+ 180°
3	Rotation about base *x*-axis	− 180°	+ 180°
4	Translation along local *x*-axis	min($$T_{x} )$$	max($$T_{x} )$$
5	Translation along local *y*-axis	min($$T_{y} )$$	max($$T_{y} )$$
6	Translation along local *z*-axis	− 200,000	min($$T_{z} )$$

### Flat-based hull

The flat-based hull RG is a modification of the conical hull, where the tip of the geometry is removed by an intersecting planar cut coincident with the base of the tumor, perpendicular to the line between the focal point and the tumor centroid. The flat-based hull is generated by rotating the bone and tumor meshes about the initial coordinate space, then applying a translation in the new local coordinates. The 3D convex hull of the tumor surface and the focal point is computed, with the focal point trimmed from the geometry and replaced with a facing cut at the lowest point of the tumor surface mesh.

The same process of rotation and translation described for the conical hull is used to generate the flat-based hull. The convex hull of the tumor mesh and the origin is computed, and all lines connecting the focal point to the tumor 3D hull are extended beyond the most distant point (tumor or bone) within the hull profile. The lines are then shortened from the focal point until the $z$ position of each connecting line is equal to the minimum tumor $z$ value, effectively removing the tip of the cone. The planar facing cut requires no additional inputs and is calculated by discarding all healthy bone voxels with a $z$ position less than the minimum tumor $z$ value in the local coordinate space. The RG is then transformed back to the initial coordinate space.

**Fig. 3:**
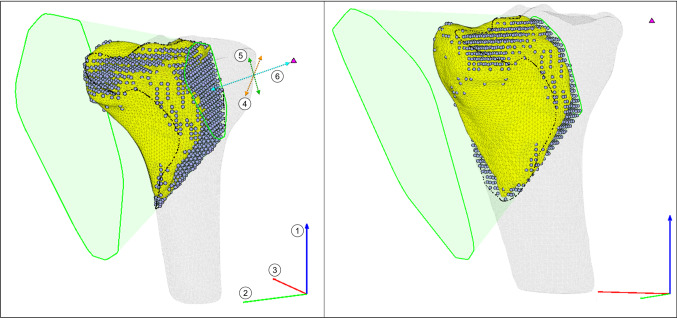
Flat-based hull resection (case 20), showing collaterally resected bone (gray circles) and RG comprised of focal point (magenta triangle), and upper and lower profiles around tumor (green splines). Base $xyz$ coordinates indicated by RGB axes, with axes of rotation (1–3). Translation in tumor local coordinate system indicated by dashed orange, green, and cyan lines (4–6)

The volume of the resection was calculated by discarding voxels with a $z$ value less than the minimum tumor $z$ value, then counting the number of healthy bone voxels within the 3D convex hull of the upper and lower sets of points, indicated by Eqs. (2) and (3),

2 $$ B_{xyz} \in {\text{inhull}}\left( {T_{xyz} ,FP} \right) $$

3 $$ B_{z} > \min \left( {T{^{\prime}}_{z} } \right) $$

where $T{^{\prime}}$ is the tumor surface mesh in its local coordinates. The RG is defined by the same input values used for the conical hull; three for the $z$, $y$, and $x$ rotations, and three for the $x$, $y$, and $z$ translation of the focal point in the rotated local coordinate system (Fig. 3). Any input between the bounds of each variable (Table 2) will produce a flat-based hull resection.

**Table 2:** Flat-based hull resection value bounds and purpose

Variable	Purpose	Lower bound	Upper bound
1	Rotation about base z-axis	− 180°	+ 180°
2	Rotation about base y-axis	− 180°	+ 180°
3	Rotation about base x-axis	− 180°	+ 180°
4	Translation along local x-axis	min($$T_{x} )$$	max($$T_{x} )$$
5	Translation along local y-axis	min($$T_{y} )$$	max($$T_{y} )$$
6	Translation along local z-axis	− 200,000	min($$T_{z} )$$

### Contoured hull

The contoured hull builds upon the flat-based hull, with the planar facing cut replaced by a curvilinear cut. The same process of rotation and translation described for the conical and flat-based hull is used in generating the contoured hull. After rotation and translation, in the new coordinate system, the tip of the cone is removed using a curvilinear cut around the 2D hull of the tumor, perpendicular to the local $z$-axis.

Compared to the conical and flat-based hull resections, an extra variable is required in the form of an extra local rotation of the bone and tumor about the local $z$-axis. Following this rotation, the 2D convex hull of the tumor is taken in the local $xz$ plane. The intersection of surfaces of the conical hull and the side profile 2D hull becomes the RG, which is transformed back to the initial coordinate space.

The volume of the resection was calculated by counting the number of healthy bone voxels within both the 3D convex hull of the conical hull, and the perpendicular 2D hull, indicated by Eqs. (4) and (5),

4 $$ B_{xz} \in {\text{inhull}}\left( {T{^{\prime}}_{xz} } \right) $$

5 $$ B_{xyz} \in {\text{inhull}}\left( {T_{xyz} ,FP} \right) $$

**Fig. 4:**
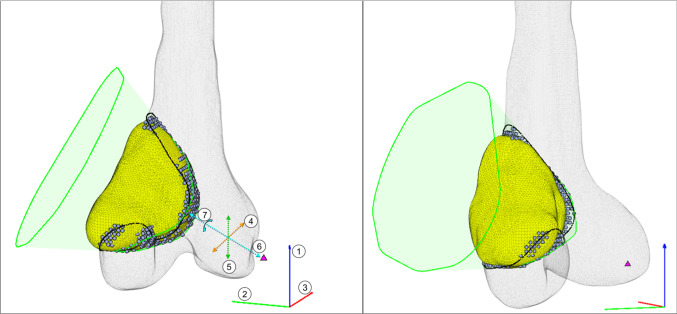
Contoured hull resection (case 13), showing collaterally resected bone (gray circles) and RG comprised of focal point (magenta triangle), and upper and lower profiles around tumor (green splines). Base $xyz$ coordinates indicated by RGB axes, with axes of rotation (1–3). Translation in tumor local coordinate system indicated by dashed orange, green, and cyan lines (4–6), and rotation about local *z*-axis (7)

The resection geometry is defined by same first six values as the conical and flat-based hulls; three for the initial $z$, $y$, and $x$ rotations, three for the $x$, $y$, and $z$ translation of the focal point in the rotated coordinate system, plus, a seventh value for local $z$ rotation (Fig. 4). Any input between the bounds of each variable (Table 3) will produce a contoured hull resection.

**Table 3:** Contoured hull resection input value bounds

Variable	Purpose	Lower bound	Upper bound
1	Rotation about base *z*-axis	− 180°	+ 180°
2	Rotation about base *y*-axis	− 180°	+ 180°
3	Rotation about base *x*-axis	− 180°	+ 180°
4	Translation along local *x*-axis	min($$T_{x} )$$	max($$T_{x} )$$
5	Translation along local *y*-axis	min($$T_{y} )$$	max($$T_{y} )$$
6	Translation along local *z*-axis	− 200,000	min($$T_{z} )$$
7	Rotation about local *z*-axis	− 90°	+ 90°

### Optimizing resection geometry via particle swarm

Particle swarm optimization (PSO) is used to compute various rotations and translations for each RG, through iterative evaluation of candidate solutions encoded in multi-variable particles moving about the problem search space. Each variable within a particle corresponds to one search value, which in this application corresponds to the specified resection geometry input variables. As a population-based stochastic optimization approach, PSO is suited to problems with a complex dimensionality or extensive search space [24].

The principle optimization metric was bone waste, equivalent to the volume of collaterally resected healthy bone with respect to the intracortical tumor volume, the same measure described in previous resection plan optimization work [10], as shown in Eq. (6).

6 $$ B_{{{\text{waste}}\% }} = \frac{{B_{{{\text{removed }}\;{\text{volume}}}} }}{{B_{{{\text{removed }}\;{\text{volume}}}} + T_{{{\text{intracortical }}\;{\text{volume}}}} }} \times 100 $$

The bounds of the particles are determined by the range of valid inputs specified by Tables 1, 2 and 3. To account for the stochastic nature of PSO and increase the likelihood of converging upon the global minimum solution, each RG was optimized 10 times, with 50 particles in the swarm. The PSO parameters utilized were default MATLAB values, with both the personal and global learning coefficients set to 1.49; an adaptive inertia based on the values of the personal and global learning coefficients for each particle, with a random initial weight for each particle between 0.1 and 1.1; and a maximum number of iterations of 200 × *n* resection parameters, with an early termination condition of 20 iterations without change.

### Manually prepared resection plans

In collaboration with an experienced surgeon, a planar resection was manually prepared for all 20 cases in a custom resection planning software. The surgeon was instructed to plan the cases assuming that the tumor surface mesh included a cuff of healthy tissue required by the principles of wide-margin tumor resection surgery, that the reconstruction could be to their choosing, and that surgical navigation would be available for all cases.

## Results

**Fig. 5:**
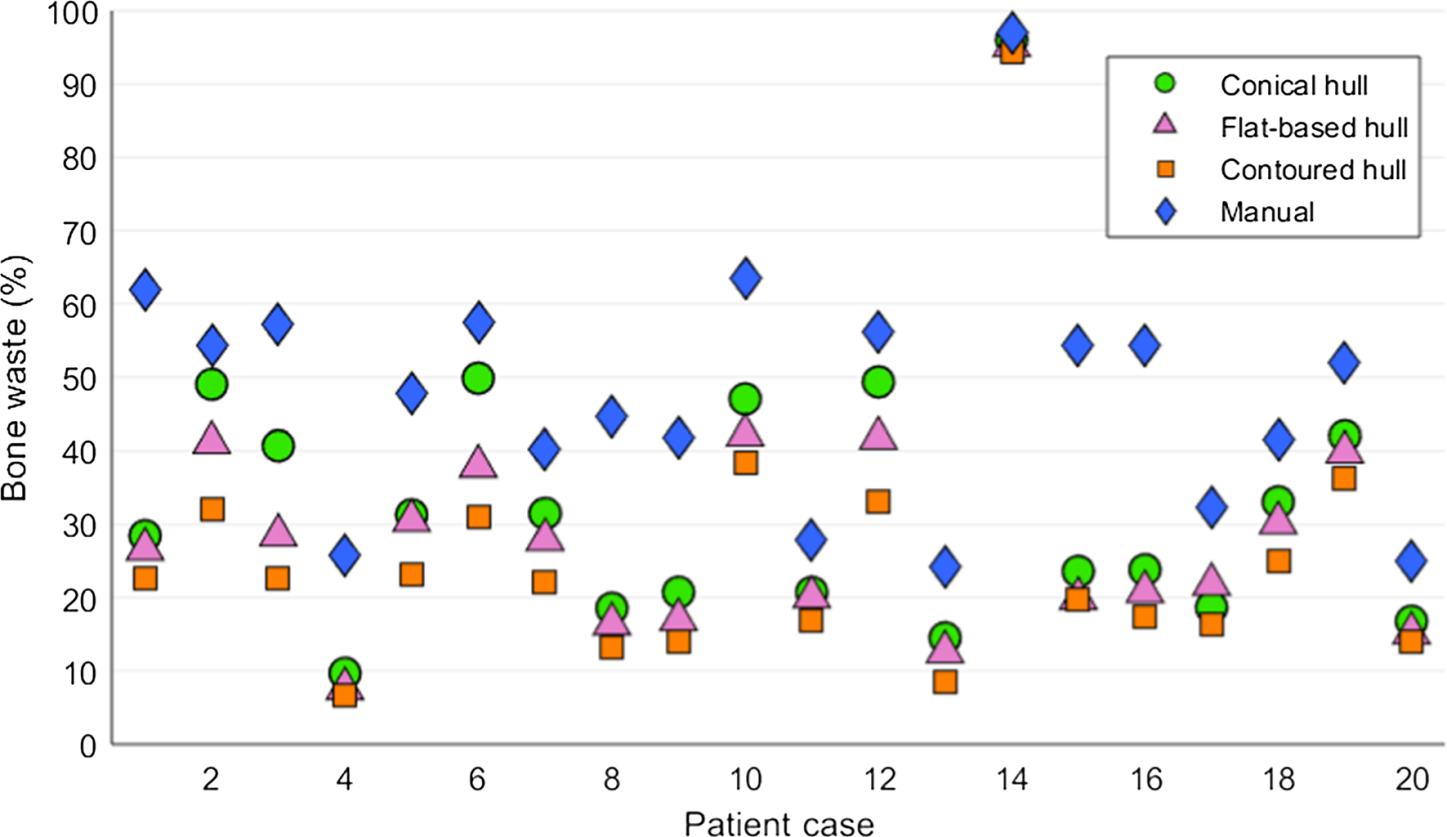
Bone waste of optimized hull-type RGs and the manual planar resection plan

The three hull-type RGs were optimized for all 20 cases. In Fig. 5, the hull-type resections are compared to the manually prepared resections. On the metric of bone waste, the hull-type resections outperformed the manual plans across all cases.

**Fig. 6:**
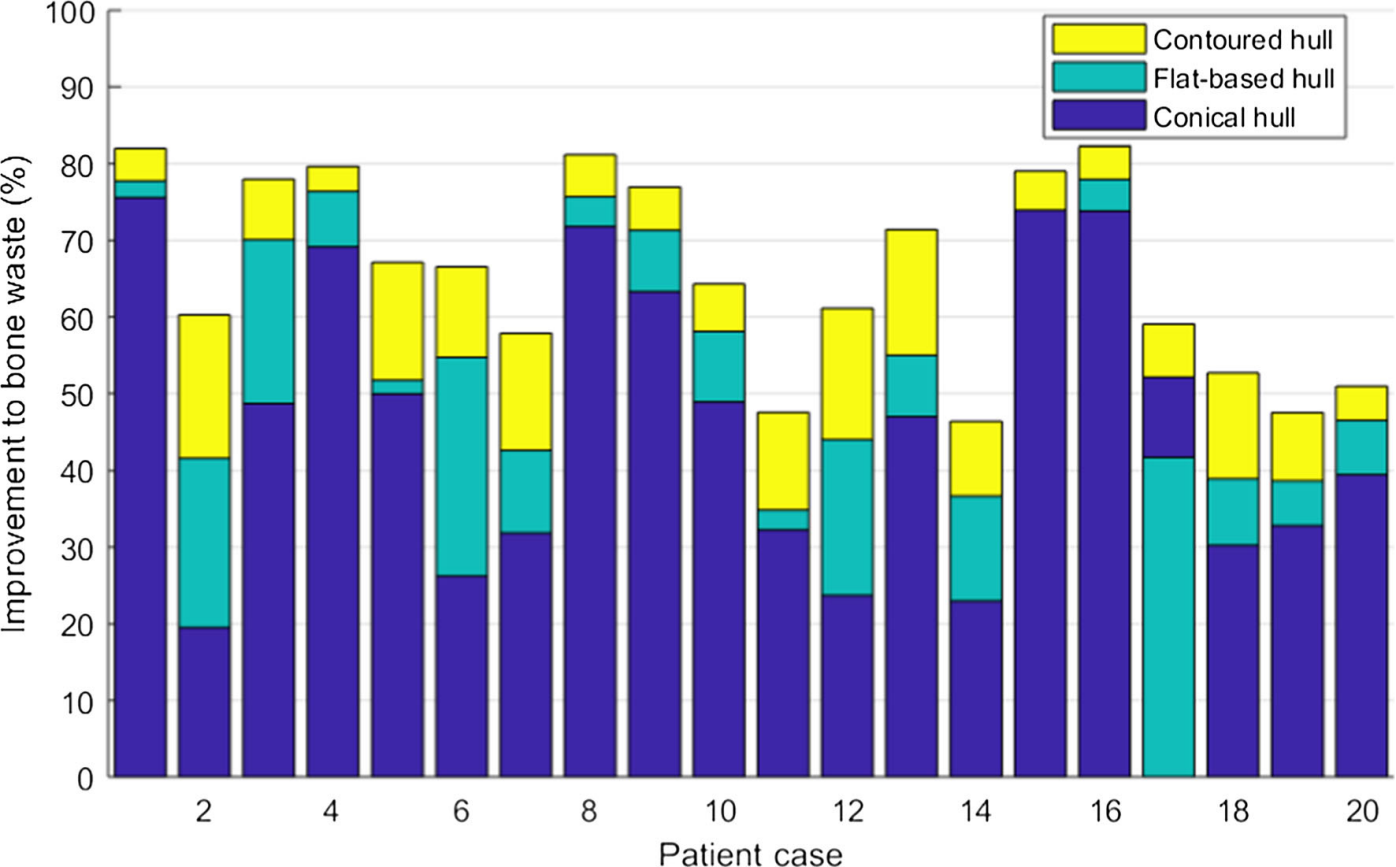
Improvement to bone waste from each RG compared to manual

Figure 6 shows the change to bone waste between the hull-type and manually prepared resections. The contoured hull had the greatest improvement for all cases, and for all but one case (case 17), the flat-based hull performed better than the conical hull.

**Fig. 7:**
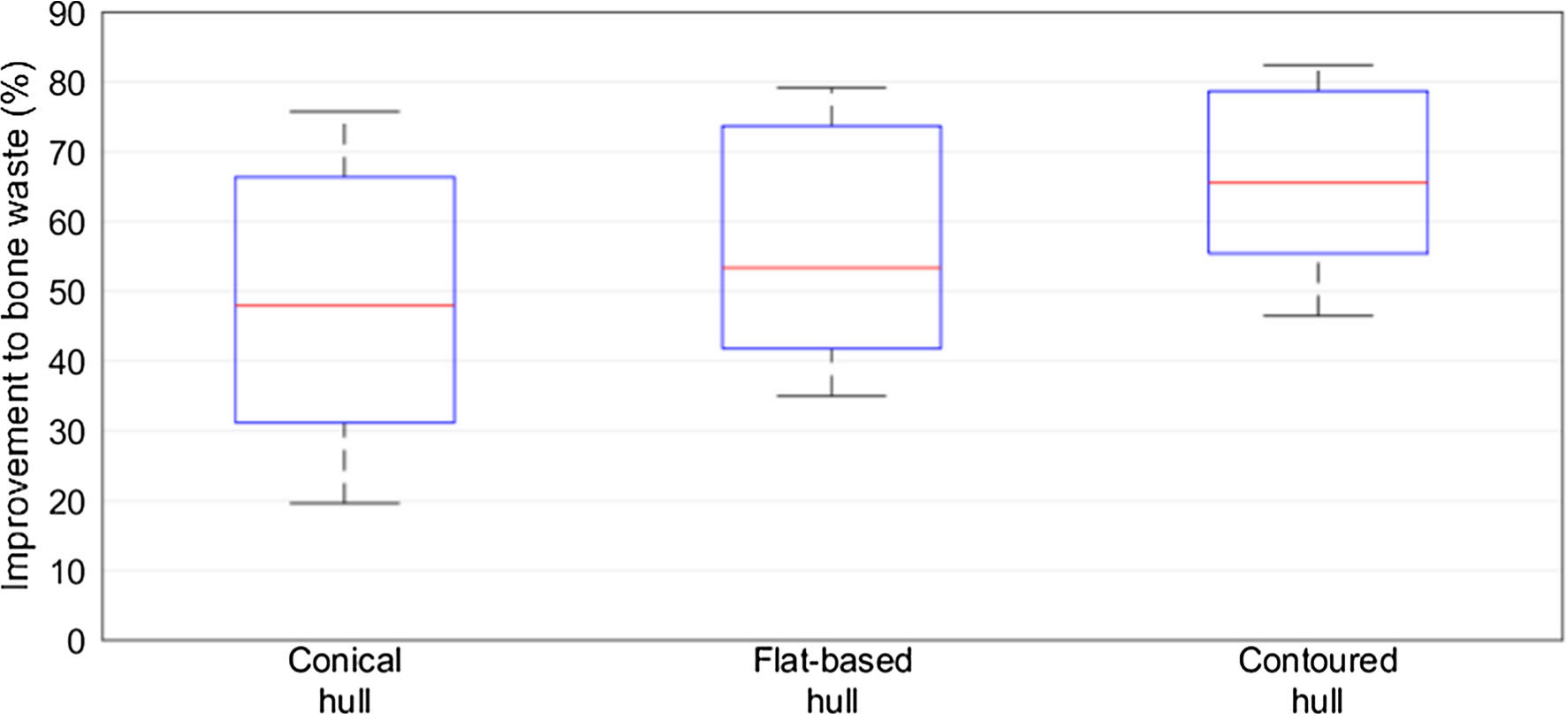
Change to bone waste compared to the manual resection

Figure 7 shows the average improvement in the elaborate RGs over planar resections. The conical hull improved upon the manual by an average of 47.82% (SD 19.14), the flat-based hull improved by an average of 53.21% (SD 16.2), and the contoured hull improved by 65.43% (SD 12.66). For all three hull-type resections, there were statistically significant improvements over manual (paired *t *test, *p* < 0.001 for each RG).

**Fig. 8:**
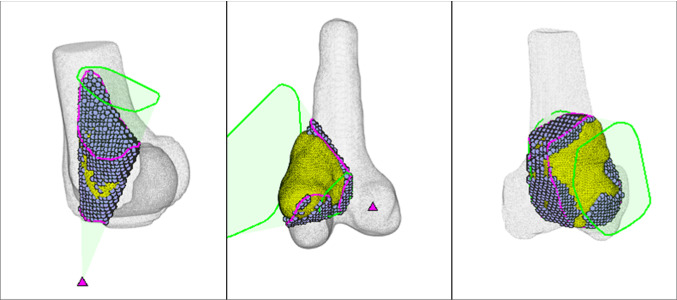
Examples of optimized conical (case 2, left), flat-based, (case 13, middle), and contoured resections (case 16, right)

Figure 8 shows the optimized resections with the minimum (19.5%), median (54.98%), and maximum (82.28%) improvements to bone waste.

**Fig. 9:**
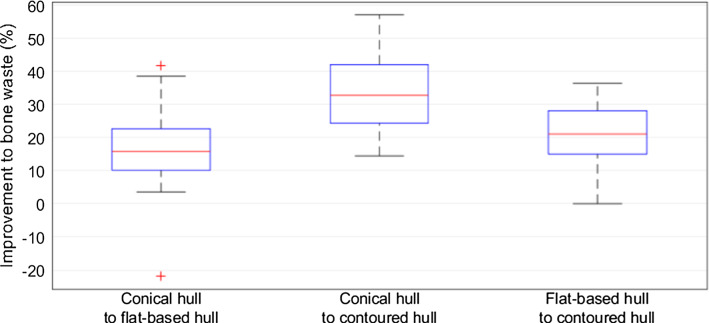
Improvement to bone waste between each hull-type RG

Figure 9 shows the change in absolute bone waste between each hull-type resection. The median improvement to bone waste between the conical and flat-based resection was 15.82% (SD 13.03), 32.82% between conical and contoured (SD 11.89), and 21.09% between flat-based and contoured (SD 8.69).

**Fig. 10:**
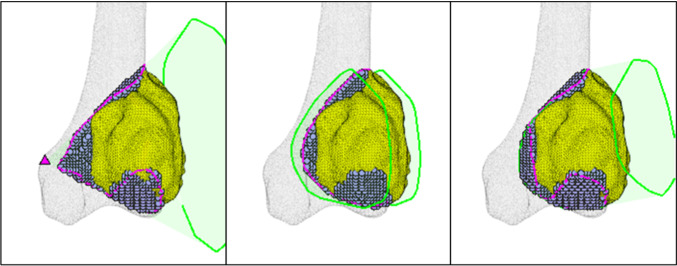
Conical, flat-based, and contoured resections for case 17

Figure 10 shows the conical, flat-based, and contoured resections for case 17, the only instance where the flat-based hull fails to improve upon the conical hull.

**Fig. 11:**
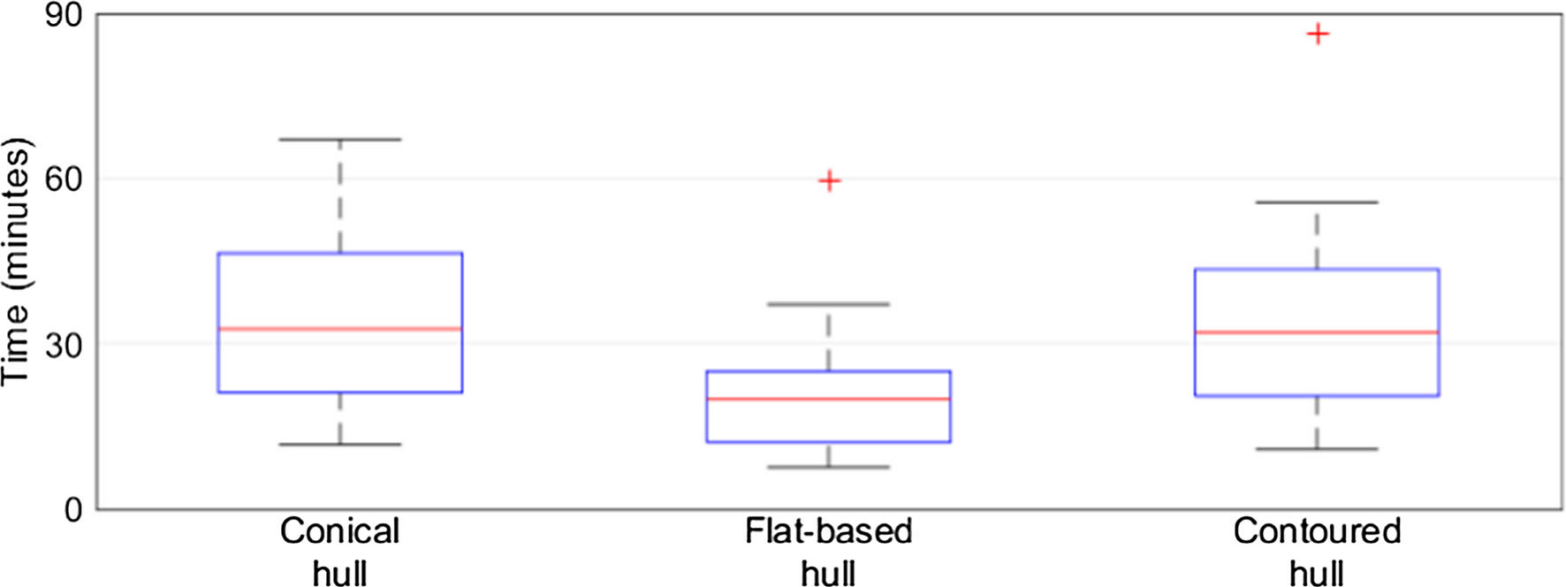
Time taken to optimize hull-type resections

Figure 11 shows the time taken to complete 10 optimizations of each hull-type resection, in minutes. The conical hull (median 32.64, SD 15.93) and contour hull (median 32.07, SD 18.27) optimized slower than the flat-base hull (median 19.80, SD 11.92).

**Fig. 12:**
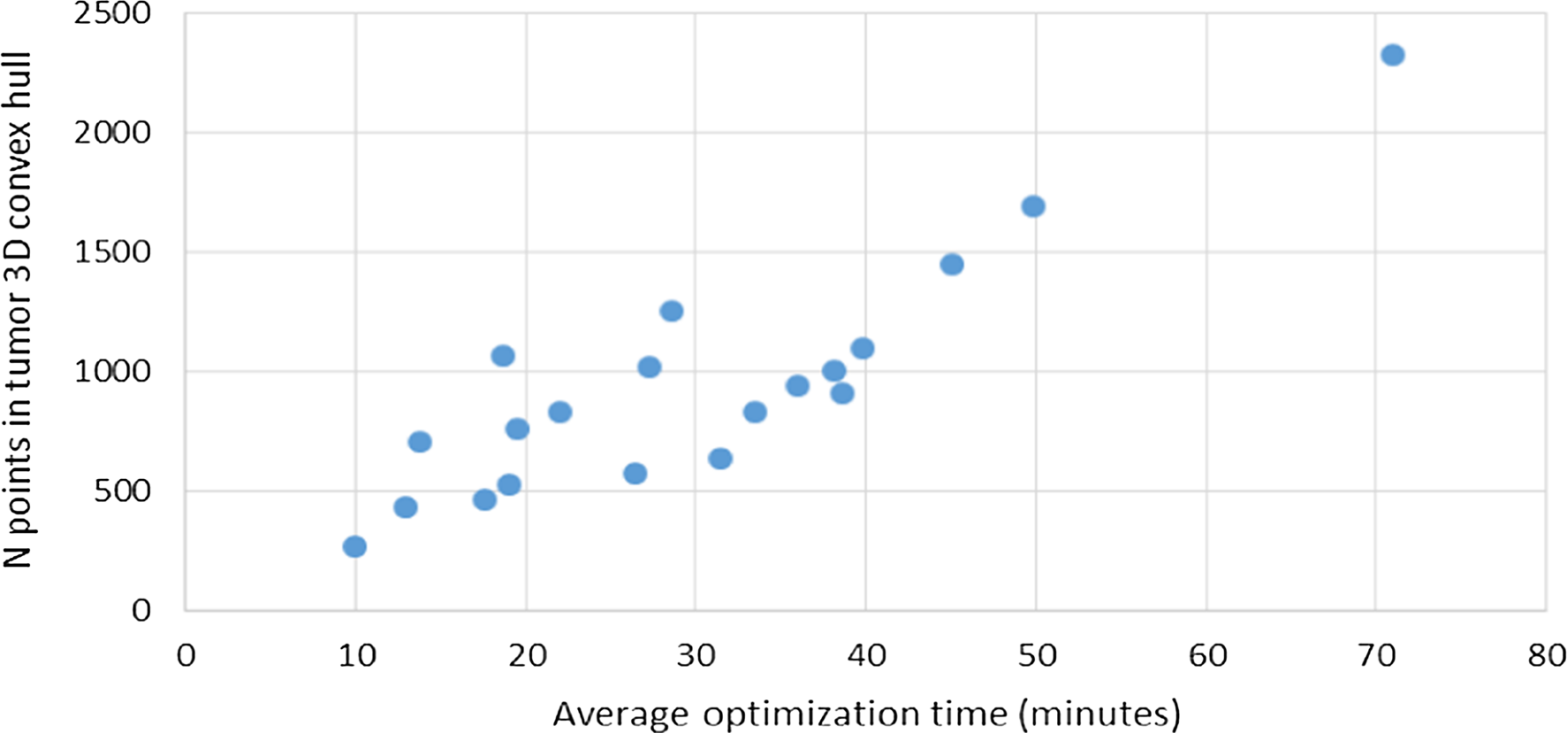
Mean optimization time for hull-type cuts compared to the number of points in the tumor hull

Figure 12 shows the mean time taken to optimize all three hull-type RGs for each case, and the number of points comprising the tumor 3D convex hull. Optimization tends to take longer where there are a greater number of points comprising the hull.

## Discussion

This work has introduced an approach for optimizing elaborate RGs which conform to a tumor volume, the results of which are compared to conventional manually prepared planar resection plans. The described approach efficiently optimizes the configuration of elaborate hull-type RGs such that the volume of healthy bone collaterally removed with the tumor is minimized. It was demonstrated that elaborate RGs based on the 3D convex hull of the tumor have less bone waste than manually prepared planar resections and that bone waste decreases as the complexity of the resection increases with additional planar and curvilinear facing cuts.

Due to the stochastic nature of PSO and the multi-dimensional search space, it is possible that a resection fails to properly optimize to a global minima. An example of this occurred in case 17 (Fig. 10) where the flat-based hull fails to improve upon the conical hull. It is possible that a candidate solution from one particle in an early iteration may have caused all other particles to prematurely converge toward an isolated region of minima, preventing the swarm from properly traversing the search space. This problem could be remedied with a greater swarm size, adjusting the PSO coefficients, or increasing the number of times each resection is optimized. Additionally, it may be possible to use the conical hull resection as an input to the other RGs, or reduce the search space based on the best conical hull resection; however, this may increase the prevalence of flat-based and contoured hull resections becoming locally optimized.

While preserving additional healthy tissue may provide functional benefits to a patient, it is more important to ensure safe margins as any diseased tissue remaining in the patient may lead to tumor recurrence. As such, defining the margin and the tissue that must be removed to completely remove the diseased tissue must be incorporated into the model. In this study the resections are generated with a margin of zero; however, this value can be adjusted by uniformly scaling the size of the tumor in 3D to include additional tissue and using the larger tumor model to define the RG. The method of defining a RG from the tumor hull and extending the conical profile to the furthest point of tissue above that geometry meant the RG could always be removed from the anatomy *en-bloc*, without the need for additional cutting operations to increase surgical access. The generated MATLAB figures and 3D images (Electronic Supplementary Material) were individually inspected to confirm that all hull-type resections could theoretically be extracted from the bone.

Although the bone waste from the elaborate RGs is typically much less than the manually prepared resection plans, the increased complexity and accuracy requirements are likely beyond the capabilities of an unassisted surgeon. In addition to surgical robots and navigation equipment, specialized cutting tools may be required to accurately perform the proposed elaborate RGs.

While the resection geometries are optimized by minimizing the volume of collaterally resected bone, the structural integrity of the remaining bone and its integration with a reconstructive prosthesis is a critical factor in long-term patient outcomes. Preservation of thin sections of bone may provide a larger surface for bone ingrowth into a prosthesis, resulting in greater long-term fixation. In the context of assessing the viability of algorithm-generated preoperative plans, the structural integrity and the expected fixation of a resection geometry could be determined by integrating finite element modeling as part of the optimization process. The inclusion of finite element analysis into the RG generation will be the subject of future work.

Each hull-type resection was optimized in under 90 min, with a mean of approximately 30 min and minimum of 10 min. From Figs. 11 and 12, the variation in optimization time is likely a result of computing the 3D convex hull of the tumor, and calculating which points of healthy bone were inside or outside the RG. The flat-based hull resections were the fastest to optimize, likely due to the Boolean removal of healthy bone points with a $z$ position less than the tumor minimum, vastly reducing the number of healthy bone points that must be checked inside the hull. Both the conical and contoured hull could be partially improved through Boolean removal of healthy bone points below the focal point. Significantly, the variations in imaging protocols across the patient scans resulted in inconsistent image resolution throughout the original data. Consequently, lower-detail tumor meshes comprised of fewer points likely optimized more quickly than high-detail tumors precisely imaged at a fine resolution.

While the optimization time may not be an argument for or against a particular RG, the treatment approach and implant manufacturing processes rely on timely generation of a viable preoperative plans. For clinicians to select and commit to a given preoperative plan, as much information as possible should be available to reduce communication bottlenecks between implant manufacture and evaluation of intraoperative process.

Despite the low incidence rate of bone tumors among the broader population, the impact of the disease upon an individual can be significant. As the unique nature of every bone tumor prohibits a one-size-fits-all treatment approach, generating a range of patient-specific surgical interventions may result in a plan which provides improved treatment outcomes and a greater quality of life.

The inherent differences across all bone tumors makes it difficult to compare not only the described approach to other methods from the literature, but also to compare the described approach on specific cases of interest or other datasets.

Quantifying and comparing a range of RGs for each tumor may show certain elaborate RGs (that might not otherwise have been considered) improve upon a conventional approach by removing less healthy bone, or preserving critical anatomy, or providing the patient with a more functional limb and greater post-operative quality of life, while still meeting the requirements of wide-margin tumor surgery. Significantly, performing these elaborate RGs will require the use of surgical robotics and other advanced operating room technologies, which further complicates the comparison to conventional approaches due to differences in surgical workflow, intraoperative duration, the precision and accuracy of tooling, and the necessity of custom implantable prostheses to reconstruct the defect.

## Conclusions and outlook

The work presented has examined an automated approach for generating elaborate resections using volumetric and surface models of anatomy. Elaborate RGs defined by the convex hull of the tumor surface mesh are optimized through particle swarm algorithms, such that bone waste is minimized. All resections are suited to *en-bloc* tumor surgery and can be adjusted to include a cuff of healthy tissue for a wide margin. Three hull-type RGs were optimized for 20 cases. Compared to the manually prepared planar resections, the hull-type resections preserved significantly more healthy bone, reducing bone waste by between 19.5% and 82.3%, with a median of 55.97%. All hull-type RGs were generated in under 90 min. The complex nature of the described geometries would require the use of surgical robots for accurate and precise intraoperative results. Future work will include various code optimizations, definition of additional elaborate RGs, testing the algorithm on tumors in more complex anatomy, and clinical evaluation of the proposed optimized resections.

## Supplementary Information

Below is the link to the electronic supplementary material.

Supplementary file1 (PDF 1332 kb)

Supplementary file2 (PDF 1935 kb)

Supplementary file3 (PDF 713 kb)

Supplementary file4 (PDF 956 kb)

Supplementary file5 (PDF 1173 kb)

Supplementary file6 (PDF 622 kb)

Supplementary file7 (PDF 1003 kb)

Supplementary file8 (PDF 955 kb)

Supplementary file9 (PDF 1281 kb)

Supplementary file10 (PDF 997 kb)

Supplementary file11 (PDF 1001 kb)

Supplementary file12 (PDF 953 kb)

Supplementary file13 (PDF 2677 kb)

Supplementary file14 (PDF 1317 kb)

Supplementary file15 (PDF 907 kb)

Supplementary file16 (PDF 833 kb)

Supplementary file17 (PDF 913 kb)

Supplementary file18 (PDF 2809 kb)

Supplementary file19 (PDF 1013 kb)

Supplementary file20 (PDF 834 kb)
