# Supplementary material for: Automated elaborate resection planning for bone tumor surgery
Source: Int J Comput Assist Radiol Surg. 2022 Nov 1;18(3):553–64. doi: 10.1007/s11548-022-02763-4 (PMC9939503; doi:10.1007/s11548-022-02763-4)
Supplement: Supplementary file 13 — Supplementary file13 (PDF 2677 kb) [file 11548_2022_2763_MOESM13_ESM.pdf]

Case 13

Document must be opened in Adobe Acrobat to enable 3D content.

To show/hide 3D elements in a figure, select 'Toggle Model Tree' in the active figure window. Each mesh can be turned on/off. For all cases, mesh N-1 and N are the bone and tumour respectively, with all other meshes allocated to cuts.

3D models can be rotated with holding left mouse button and moving mouse. Zoom in / out is performed by holding right mouse button and moving mouse forward / backward, or using mouse wheel. Model can be translated in 3D space by moving mouse while holding both ctrl and left mouse button.

Initial state

Manually prepared resection plan

Conical hull resection

Flat-based hull resection

Contoured hull resection
